# Should Aspergillus screening before bronchial thermoplasty?

**DOI:** 10.1186/s12890-021-01536-0

**Published:** 2021-06-09

**Authors:** Shinji Sasada

**Affiliations:** Department of Respiratory Medicine, Tokyo Saiseikai Central Hospital, 1-4-17 Mita, Minato-ku, Tokyo 108-0073 Japan

**Keywords:** Allergic bronchopulmonary Aspergillosis, Bronchial thermoplasty, Severe asthma

## Main text

Thank you for your feedback. If there were any findings suspicious of allergic bronchopulmonary Aspergillosis (ABPA), aspergillus-specific antibody measurements and skin tests would have been performed as screening [1]. However, this case did not show any findings considered to be ABPA such as bronchodilation and mucus plugs on computed tomography (CT) and bronchoscopy before treatment [2]. On the other hand, regarding the sessions of bronchial thermoplasty (BT) for severe asthma, the third session was performed after the sample was collected, so BT treatment was completed. Regarding BT indications, although regular steroid administration was not performed in this case, a single rescue steroid administration was performed, and it is judged to be within the indication in Japan.

The question is, do you order Aspergillus-specific antibodies or skin tests on all cases of asthma that are not abnormal on CT and bronchoscopy?

## Data Availability

All data and material are available for sharing if needed.

## Abbreviations

ABPA: Allergic bronchopulmonary Aspergillosis
BT: Bronchial thermoplasty
CT: Computed tomography
